# A Scoring System with High-Resolution Computed Tomography to Predict Drug-Associated Acute Respiratory Distress Syndrome: Development and Internal Validation

**DOI:** 10.1038/s41598-019-45063-9

**Published:** 2019-06-13

**Authors:** Keisuke Anan, Kazuya Ichikado, Takuma Ishihara, Ayumi Shintani, Kodai Kawamura, Moritaka Suga, Takuro Sakagami

**Affiliations:** 1 Division of Respiratory Medicine, Saiseikai Kumamoto Hospital, Kumamoto, Japan; 2 Innovative and Clinical Research Promotion Center, Gifu University Hospital, Gifu, Japan; 3 Department of Medical Statistics, Osaka City University Graduate School of Medicine, Osaka, Japan; 4 Department of Respiratory Medicine, Kumamoto University Hospital, Faculty of Life Sciences, Kumamoto University, Kumamoto, Japan

**Keywords:** Diagnosis, Respiratory distress syndrome

## Abstract

Drugs can cause acute respiratory distress syndrome (ARDS). However, there is no established clinical prediction rule for drug-associated ARDS (DARDS). We aimed to develop and validate a scoring system for DARDS prediction. We analysed data collected from a prospective, single-centre, cohort study that included ARDS patients. The ARDS diagnosis was based on the American-European Consensus Conference or Berlin definition. Drug-associated acute lung injury (DALI) was defined as previous exposure to drugs which cause ALI and presence of traditional risk factors for ALI. High-resolution computed tomography (HRCT; indicating extent of lung damage with fibroproliferation), Acute Physiology and Chronic Health Evaluation (APACHE) II, and disseminated intravascular coagulation (DIC; indicating multiorgan failure) scores and PaO_2_/FiO_2_ were evaluated for their ability to predict DARDS. Twenty-nine of 229 patients had DARDS. The HRCT, APACHE II, and DIC scores and PaO_2_/FiO_2_ were assessed. The model-based predicted probability of DARDS fitted well with the observed data, and discrimination ability, assessed through bootstrap with an area under the receiver-operating curve, improved from 0.816 to 0.875 by adding the HRCT score. A simple clinical scoring system consisting of the APACHE II score, PaO_2_/FiO_2_, and DIC and HRCT scores can predict DARDS. This model may facilitate more appropriate clinical decision-making.

## Introduction

Acute respiratory distress syndrome (ARDS) is a life-threatening manifestation of acute lung injury, and the associated mortality remains high^1^ despite developments in modern medicine. ARDS reportedly has various clinical phenotypes with different risks and prognostic factors^2–4^. Moreover, certain drugs have been reported to cause ARDS^5,6^. Although diagnostic criteria for drug-associated lung injury (DALI) have been proposed^7,8^, there is still no gold standard and its diagnosis remains challenging. It is particularly difficult to diagnose DALI in patients with ARDS.

Recently, we reported that drug-associated ARDS (DARDS) has clinical characteristics that are different from those of non-drug-associated ARDS (non-DARDS) and that DARDS has a more favourable prognosis^9^. In that study, patients with DARDS had a lower Acute Physiology and Chronic Health Evaluation (APACHE) II score and a higher arterial oxygen tension (PaO_2_)/fractional inspired oxygen (FiO_2_) ratio than those without DARDS. However, high-resolution computed tomography (HRCT) scans showed more extensive lung damage and fibroproliferation in the patients with DARDS. Furthermore, another retrospective cohort study reported that patients with ARDS, who did not have typical risk factors, including those with DARDS, were more frequently referred to the intensive care unit for acute respiratory failure, had a lower severity score on admission to the intensive care unit, and were less likely to develop non-pulmonary organ failure^10^.

Although corticosteroid therapy for ARDS is controversial, it is generally used for DALI. There may be a therapeutic advantage of using steroids in patients with DARDS if they could be diagnosed. We believe that we should consider the clinical features and treatment strategy used for DARDS separately from that of non-DARDS because the effect of treatment, e.g., with corticosteroids, and the prognosis of these disease entities may be different. We hypothesized that it would be possible to use a scoring system to predict drug-associated DARDS using clinical variables. The purpose of this study was to develop and validate a scoring system for prediction of DARDS.

## Results

**Figure 1:**
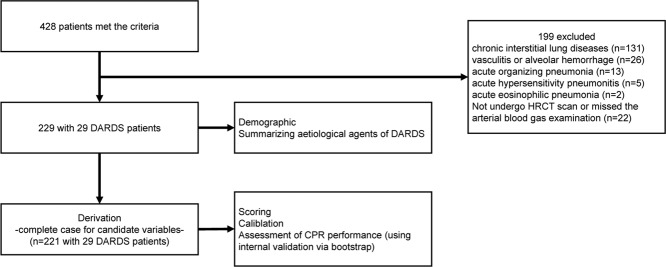
Flow of patients through the study. CPR, clinical prediction rule; HRCT, high-resolution computed tomography.

There were 29 patients in the DARDS group and 200 in the non-DARDS group (Fig. 1). The baseline characteristics of the patients in the two groups are shown in Table 1.

**Table 1:** Baseline characteristics of patients in the DARDS and non-DARDS groups

	DARDS group (n = 29)	Non-DARDS group (n = 192)	*p*-value
	Probable DARDS (n = 22)	Conditional DARDS or without DARDS (n = 192)	
	Possible DARDS (n = 5)		
Age (years)	75.0 [72.0–79.0]	75.5 [67.0–83.0]	0.99
Male sex	12 (41)	127 (66)	0.01
White blood cell count (per mm^3^)	12,000 [9500–15,100]	10200 [5375–14,650]	0.152
Lactate dehydrogenase (IU/l)	475 [358–579]	313 [243–451]	<0.001
Platelet count (per mm^3^)	20.6 [12.5–29.8]	18.0 [11.3–24.6]	0.123
C-reactive protein (mg/dl)	15.3 [13.4–21.0]	16.0 [8.9–25.3]	0.654
Albumin (g/dl)	2.9 [2.6–3.1]	2.8 [2.4–3.2]	0.804
APACHE II score	18.0 [16.0–21.0]	22.0 [18.0–25.3]	<0.001
SOFA score	5.0 [3.0–7.0]	7.0 [5.0–10.3]	0.001
HRCT score	301.6 [248.2–343.0]	211.4 [183.4–277.0]	<0.001
PaO_2_/FiO_2_	148.0 [121.0–179.5]	106.4 [74.2–140.3]	0.001
DIC score			0.167
0	0 (0)	8 (4)	
1	8 (28)	35 (18)	
2	12 (41)	47 (25)	
3	5 (17)	31 (16)	
4	3 (10)	29 (15)	
5	0 (0)	24 (13)	
6	0 (0)	7 (4)	
7	1 (4)	11 (6)	
McCabe score (1/2/3)	21 (72)/5 (17)/3 (10)	165 (86)/14 (7)/13 (7)	0.143

The data are shown as the number (percentage) or median [interquartile range]. APACHE II, Acute Physiology and Chronic Health Evaluation II; ARDS, acute respiratory distress syndrome; DARDS, drug-associated acute respiratory distress syndrome; DIC, disseminated intravascular coagulation; HRCT, high-resolution computed tomography; SOFA, Sequential Organ Failure Assessment.

The aetiological agents associated with DARDS are shown in Table 2. The causative agents were anticancer drugs in 7 cases (24%), Chinese herbal (“kampo”) medicine in 5 (17%), antibiotics in 4 (14%), amiodarone in 4 (14%), anti-rheumatic agents in 3 (10%), non-steroidal anti-inflammatory drugs in 3 (10%), and others in 3 (10%).

**Table 2:** Agents known to cause drug-associated acute respiratory distress syndrome identified in 29 patients.

DARDS n = 29	
Probable (n = 24, 83%)	
Possible (n = 5, 17%)	
Agents	n (%)
**Anti-neoplastic**	7 (24)
CHOP	2
Gefitinib	1
Irinotecan	1
Bicalutamide	1
Docetaxel	1
Epirubicin	1
**Chinese herbal medicine**	5 (17)
Seishin-renshi-in	3
Juncho-to	1
Toki-kenchu-to	1
**Antibiotic**	4 (14)
Cephalosporin	2
Penicillin	1
Daptomycin	1
**Anti-arrhythmic**	
Amiodarone	4 (14)
**Anti-rheumatic**	3 (10)
**Non-steroidal anti-inflammatory**	3 (10)
**Novel oral anticoagulant**	1 (3)
**Antiviral**	
Daclatasvir and asunaprevir	1 (3)
**Dipeptidyl peptidase 4 inhibitor**	1 (3)

CHOP, cyclophosphamide, doxorubicin, vincristine, prednisolone; DARDS, drug-associated acute respiratory distress syndrome.

**Figure 2:**
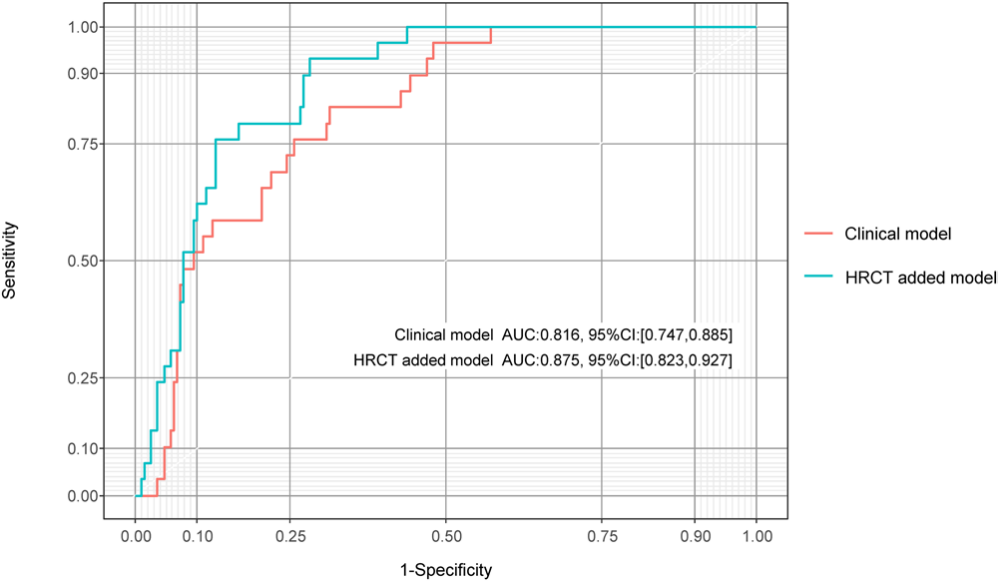
Area under the receiver-operating characteristic curve for each model.

Clinical prediction rule was developed using 2 multivariable logistic regression models: a model that included the APACHE II score, DIC score, and PaO_2_/FiO_2_ ratio; and a model in which the HRCT was added to the APACHE II score, DIC score, and PaO_2_/FiO_2_ ratio. The receiver-operating characteristic (ROC) curves for the clinical and HRCT added models are shown in Fig. 2 and the area under the ROC curve (AUC-ROC), bootstrap AUC-ROC, and optimism in Table 3.

**Table 3:** AUC-ROC, bootstrap AUC-ROC, and optimism values in the multivariable logistic regression models.

	AUC-ROC	95% CI	Bootstrap AUC-ROC	Optimism
Clinical model	0.816	0.747–0.885	0.796	0.265
HRCT added model	0.875	0.823–0.927	0.850	0.247

Clinical model, APACHE II score, DIC score, and PaO_2_/FiO_2_ ratio; HRCT added model, APACHE II score, DIC score, HRCT score, and PaO_2_/FiO_2_ ratio. APACHE II, Acute Physiology and Chronic Health Evaluation II; AUC, area under the curve; CI, confidence interval; DIC, disseminated intravascular coagulation; HRCT, high-resolution computed tomography; ROC, receiver-operating characteristic curve.

**Figure 3:**
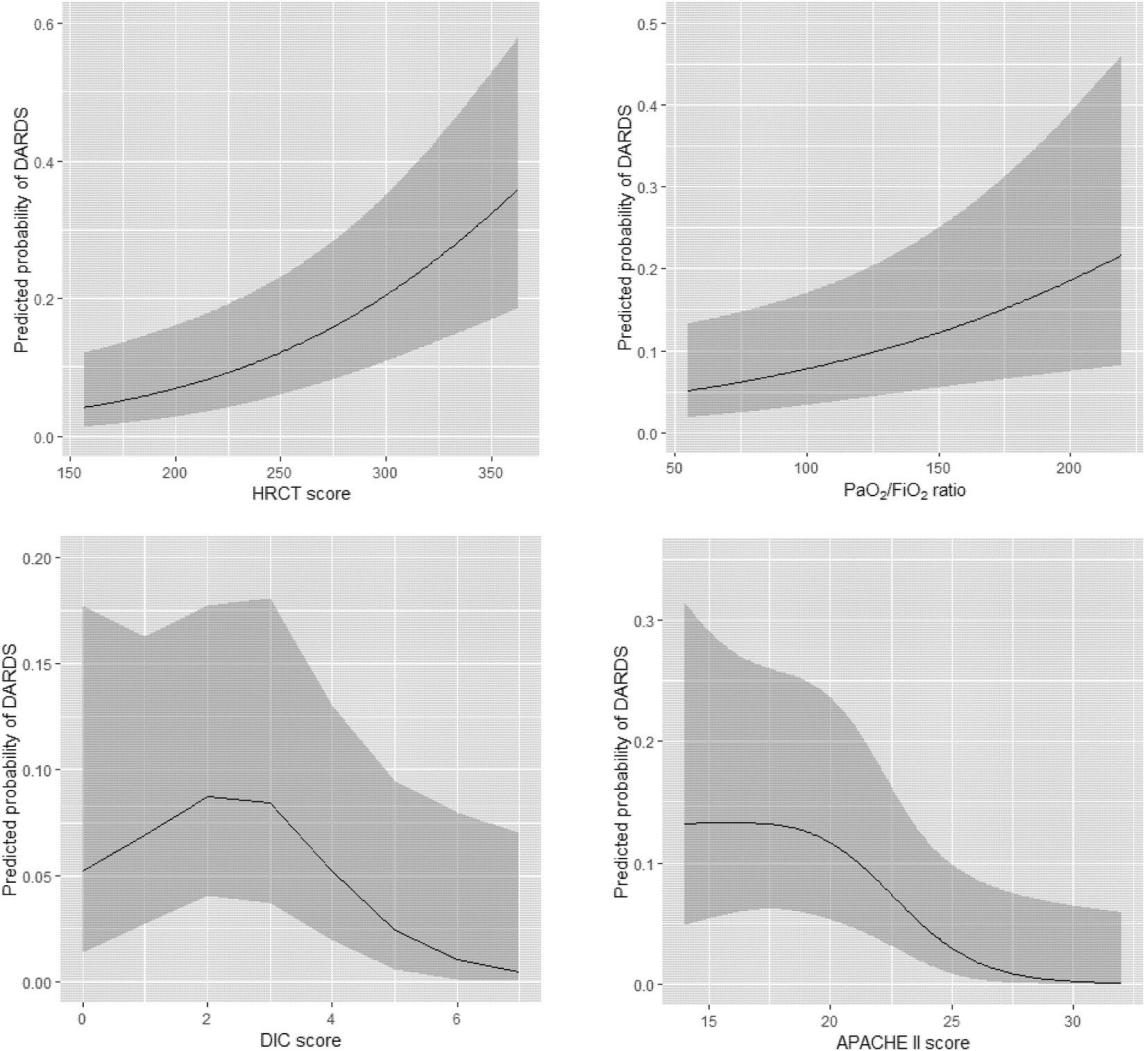
The relationship between DARDS and each variable in the HRCT added model.

**Figure 4:**
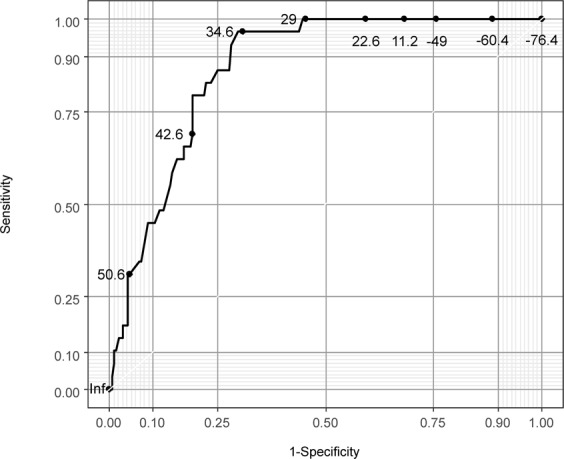
Area under the receiver-operating characteristic curve for the final model.

We assessed the additive predictive ability of HRCT in diagnostic ability between the clinical model and HRCT added model using net reclassification improvement (NRI) and integrated discrimination improvement (IDI; Table 4) and both were statistically significant (P < 0.01). We found that adding HRCT appeared to be superior to the model including only the clinical variables. The relationship between the predicted probability of DARDS and each continuous variable are shown in Fig. 3. Next, some of the variables in the HRCT added model were categorized according to their ease of use in the clinical setting (Table 5) to create a clinical prediction rule for diagnosing DARDS. The ROC curve for the final model is shown in Fig. 4. The AUC-ROC was 0.866 (95% confidence interval 0.814–0.920). The bootstrap method was used to evaluate the internal validity of the final model. The AUC-ROC was 0.853 when adjusted for optimism, which was 0.136.

**Table 4:** Additive diagnostic ability of DARDS with a model including HRCT score compared with a model including APACHE II score, DIC score, and PaO_2_/FiO_2_ ratio using net reclassification improvement (NRI) and integrated discrimination improvement (IRI).

	Total	95% CI	*p*-value
NRI	0.717	0.348–1.086	<0.01
IDI	0.084	0.031–0.137	<0.01

The total value for NRI or IDI was computed compared with Clinical and HRCT added models to predict DARDS. Clinical model, APACHE II score, DIC score, and PaO_2_/FiO_2_ ratio; HRCT added model, APACHE II score, DIC score, PaO_2_/FiO_2_ ratio, and HRCT score. APACHE II, Acute Physiology and Chronic Health Evaluation II; CI, confidence interval; DIC, disseminated intravascular coagulation; HRCT, high-resolution computed tomography; IDI, integrated discrimination improvement; NRI, net reclassification improvement; SOFA, Sequential Organ Failure Assessment.

**Table 5:** Variables in the clinical prediction rule.

Variable	Category	Score
HRCT score	integer number	0.122 × integer number
APACHE II score	≤19	0
	20−24	−4.4
	≥25	−89
DIC score	0−3	0
	≥4	−11.4
PaO_2_/FiO_2_ ratio	integer number	0.094 × integer number

APACHE II, Acute Physiology and Chronic Health Evaluation II; DIC, disseminated intravascular coagulation; HRCT, high-resolution computed tomography.

Using a potential cut-off score of >34.8, the sensitivity was 96.6%, the specificity was 70.3%, the positive predictive value was 32.9%, and the negative predictive value was 99.3%.

## Discussion

In this study, we have developed a clinical prediction rule for DARDS in patients with a known diagnosis of ARDS. There are four variables in this clinical prediction rule, which we have named the “DHAP” (DIC score, HRCT score, APACHE II score, PaO_2_/FiO_2_) score. Using this clinical prediction rule, the higher the DIC and APACHE II scores the lower the DHAP score, and the higher the HRCT score and PaO_2_/FiO_2_ ratio the higher the DHAP score. The Japanese Association for Acute Medicine has proposed a DIC scoring system that reportedly predicts multiorgan failure and a poor prognosis in patients with severe sepsis^11^. There is a correlation between the findings on HRCT findings and the pathologic stage of diffuse alveolar damage (DAD) as well as an association of HRCT scores with a poor outcome, prolonged mechanical ventilation, multiple organ dysfunction, and ventilator-associated complications (barotrauma and ventilator-associated pneumonia)^12^. In view of the above findings, the clinical prediction rule devised in the present study suggests that extensive lung damage with fibroproliferation and a less serious general and respiratory condition are important indicators of DARDS.

A DHAP score >34.8 had high sensitivity and a negative predictive value. It also had a high AUC-ROC and bootstrap AUC-ROC, and its optimism was reasonable.

ARDS has recently been reported to include a variety of clinical phenotypes with divergent risk factors and prognostic features^2–4^. In those studies, the response of the hyperinflammatory sub-phenotype, characterized by an increase in levels of biomarkers of inflammation, more severe shock and acidosis, and a significantly poorer prognosis, to positive end-expiratory pressure, fluid therapy, and pharmacotherapy was found to be different to that of the hypoinflammatory sub-phenotype. As already mentioned, ARDS is a heterogeneous syndrome, and it would be helpful to be able to categorize it according to its cause and tailor treatment to the sub-phenotype.

In previous reports, DALI/DARDS accounted for about 10% of all cases of ALI/ARDS^7,9,10^. Recently, we reported that the clinical features of DARDS were different from those of non-DARDS^9^. In that study, patients with DARDS received higher doses of corticosteroids and had a better prognosis than those with non-DARDS. Moreover, Gibelin *et al*.^10^ reported that the cytology of bronchoalveolar lavage (BAL) fluid and findings on chest CT might help to detect patients with ARDS, including those with drug-induced ARDS, who do not have the common risk factors, and that their mortality risk might be decreased by treatment with an anti-inflammatory agent.

In Japan, the Practical Guidelines of the Japanese Respiratory Society^13^ recommend that patients with acute respiratory failure caused by DALI be treated initially with high-dose corticosteroids and gradually tapered. It is possible that steroid therapy could be beneficial in patients with DARDS if they could be identified.

Histopathologic features of DAD were reportedly found at autopsy in only 45% of patients with ARDS diagnosed by the Berlin criteria^14^. This suggests that not all patients with ARDS have the histopathologic features of DAD. One possible explanation for this observation is that there might be some pathologic differences in patients with DARDS who develop fibroproliferative changes, and corticosteroid therapy could have a better effect in DARDS.

It was reported that patients with ARDS and predominantly haemorrhagic or lymphocytic BAL fluid had a better response to corticosteroid therapy and a better outcome than those with predominantly macrophagic or neutrophilic BAL fluid^10^. Recently, we reported that 62% of patients with DARDS in whom BAL was performed had lymphocytic or eosinophilic BAL fluid^9^. These findings suggest that many patients with DARDS have lymphocytic BAL fluid and that corticosteroid therapy may be effective in these patients. The value of corticosteroid therapy for ARDS remains controversial. If corticosteroids are withheld in all patients with ARDS, those with DARDS may miss the opportunity for potentially effective treatment. DARDS cannot be diagnosed definitively using the DHAP score alone. However, when combined with BAL and lymphocyte predominance, this score may help to diagnose DARDS. We believe that it is important to be able to determine whether ARDS is drug-associated and that the DHAP score can help to make the diagnosis.

There are some limitations to this study. First, it had a single-centre, retrospective design, which may limit use of the DHAP score at other facilities. Further prospective multicentre validation studies are necessary to determine if use of this tool could be extended to a more general patient population. Second, the number of patients with DARDS was relatively small, so our clinical prediction rule may have the problem of overfitting. However, the accuracy of this rule was sufficiently high, and the optimism of the final model was adequate. Third, only Japanese patients participated in the study, and we may have to consider ethnic differences when using this rule. Fourth, it was difficult to judge whether some drugs were causative or not in the DARDS group. However, this problem is unavoidable in such research because specific markers, histologic features, and clinical findings are generally not diagnostic, and there is no gold standard method for diagnosis of drug-associated lung disease. Fifth, the DHAP score includes the HRCT score, which is not used worldwide and is only used when a patient with ARDS has recently been exposed to a suspected culprit drug.

In conclusion, we have developed a simple scoring system for clinical prediction of DARDS. We believe that using this rule will help early detection, guide appropriate treatment, and improve the prognosis in patients with DARDS. A further external validation study for this clinical prediction rule is now needed.

## Methods

### Study design

This study was a post-hoc analysis of data collected during an ongoing prospective, single-centre, cohort study of ARDS using HRCT, parts of which have been published previously^9,12,15–18^. Our hospital is a tertiary academic teaching institution with 400 beds.

### Patients

Two hundred and twenty-nine Japanese patients with ARDS were admitted to our institution from October 2004 to December 2017. The ARDS diagnosis was based on the American-European Consensus Conference^19^ before June 2012 and on the Berlin definition^20^ after July 2012. The study participants were enrolled from both the intensive care unit and other departments within the hospital. An HRCT scan was performed on the day of diagnosis of ARDS in all cases. Patients with chronic interstitial lung disease, including idiopathic pulmonary fibrosis and underlying collagen vascular disease, and those with conditions that mimic ARDS (pulmonary alveolar haemorrhage resulting from vasculitis syndrome, acute organizing pneumonia, acute eosinophilic pneumonia, and acute hypersensitivity pneumonitis) were excluded from the analysis.

The study was approved by the institutional review board of Saiseikai Kumamoto Hospital (permission number 238) and conducted in accordance with the ethical standards of the Declaration of Helsinki. Written informed consent was obtained from all patients included in the study or their families. The results of the study are reported in accordance with the Transparent Reporting of a multivariable prediction model for Individual Prognosis Or Diagnosis (TRIPOD) statement^21^.

### Definition of drug-associated ARDS

We classified the patients with ARDS into a DARDS group and a non-DARDS group according to aetiology. We used the definition of DALI devised by Dhokarh *et al*.^7^ and the traditional risk factors for acute lung injury (ALI), i.e., sepsis, septic shock, pneumonia, pancreatitis, trauma, massive blood transfusion, and gastric aspiration. Probable DALI was considered in patients with no established risk factors for ALI except for specific drug exposure within the previous year. Patients with possible DALI had at least one risk factor for ALI and a history of specific drug exposure within the previous year. Those with conditional DALI had received drugs not previously reported to cause ALI but with similarity to known causative agents. Patients without DALI had not been exposed to drugs reported or assumed to cause ALI. The patients with ARDS and probable or possible DALI were classified into a DARDS group while those with ARDS and conditional DALI or without DALI were classified into a non-DARDS group. The data for drug exposure history in the year before onset of ARDS were available in “medicine notebooks” that list all drugs prescribed to patients and are unique to Japan.

### Data collection and definitions

Patient age and sex, APACHE II score, Sequential Organ Failure Assessment (SOFA) score, HRCT score (indicating the extent of fibroproliferation^12^), DIC score, McCabe score, arterial oxygen tension (PaO_2_/FiO_2_) ratio, and blood test results were recorded at the time of diagnosis of ARDS. HRCT was performed at the start of mechanical ventilation in all patients. As previously reported^22^, the HRCT score was graded on a scale of 1−6 (1, normal attenuation; 2, ground-glass attenuation; 3, consolidation; 4, ground-glass attenuation with traction bronchiolectasis or bronchiectasis; 5, consolidation with traction bronchiolectasis or bronchiectasis; and 6, honeycombing). The presence of each of these six abnormalities in the upper, middle, and lower segments of each lung was assessed independently. The extent of each abnormality was determined by visually estimating the proportion of lung parenchyma affected in each segment. Each abnormality score was calculated by multiplying the percentage area by each individual score. The six segment scores were averaged to determine the total score for each abnormality. The overall HRCT score was obtained by adding the six averaged scores.

The DIC score was calculated according to the diagnostic criteria recommended by the Japanese Association of Acute Medicine^23^. The McCabe score for severity of underlying disease was recorded (1, non-fatal; 2, near-fatal; 3, fatal)^24^.

### Statistical analysis

Continuous variables are described as the median (interquartile range [IQR]) and the categorical variables as the frequency and percentage. The chi-square test or Fisher’s exact test was used to compare the categorical variables between the groups and the Mann-Whitney *U* test was used to compare the continuous variables between patients with and without DARDS. Variables with clinical relevance, including the APACHE II score, DIC score, HRCT score, and PaO_2_/FiO_2_ ratio, were selected a priori and included in multivariable logistic regression models where the binary variable of present or absence of DARDS was the dependent variable. We created 2 models for development of a clinical prediction rule for DARDS: a clinical model consisting of the APACHE II score, DIC score, and PaO_2_/FiO_2_ ratio; and an HRCT added model consisting of the APACHE II score, DIC score, PaO_2_/FiO_2_ ratio, and HRCT score. To allow for nonlinear associations, the APACHE II and DIC scores were modelled using restricted cubic splines.

Given that the number of patients with missing data was small (n = 8), the prediction model was developed using data only for patients in whom all of the study variables had been assessed (n = 221). The discrimination performance of each potential predictor was assessed by the AUC-ROC. Bootstrap validation was performed with 150 resamples to validate and calibrate each prediction model. The bootstrap bias-corrected AUC (bootstrap AUC-ROC) was reported as the measure of the predictive performance of the model. The optimism^25^ of each model was estimated using 150 bootstrap resamples. Optimism assesses the magnitude of overfitting of logistic regression model (a value less than 0.3 is considered as good), and was calculated using C-statistics by bootstrap samples. We evaluated the bootstrap AUC-ROC and overfitting of each model and chose two parsimonious models with acceptable diagnostic ability and the least number of parameters. Using the NRI and IDI, we assessed whether there was a difference in diagnostic ability between the 2 models. The total NRI was the summation of the accurate reclassifications of patients with and without DARDS. In the patients with DARDS, improvement of reclassification was the difference between the percentage of patients reclassified as a higher risk group and that of patients reclassified as a lower group. Similarly, in the patients without the DARDS, improvement of reclassification was the difference between the percentage of patients reclassified as a lower risk group and that of patients reclassified as a higher group. The total IDI provides the difference in mean predicted probabilities, representing the amount by which addition of a variable to a model increases the separation of the mean predicted probabilities for DARDS and non-DARDS^26^. To make the final model easier to use in a clinical setting, the variables were scored, the bootstrap AUC-ROC of the final model was calculated, and its diagnostic ability was assessed. The sensitivity, specificity, positive predictive value, and negative predictive value were calculated by using the best cut-off score for the clinical prediction rule with the Youden index for the ROC.

The statistical analyses were performed using R version 3.5.1 (R Foundation for Statistical Computing, Vienna, Austria). A two-sided *p*-value < 0.05 was considered statistically significant.

## References

[1] Thompson BT, Chambers RC, Liu KD (2017). Acute respiratory distress syndrome. N. Engl. J. Med..

[2] Calfee CS (2018). Irish Critical Care Trials Group. Acute respiratory distress syndrome subphenotypes and differential response to simvastatin: secondary analysis of a randomized controlled trial. Lancet Respir. Med..

[3] Calfee CS (2015). Distinct molecular phenotypes of direct vs indirect ARDS in single-center and multicenter studies. Chest.

[4] Calfee CS (2014). NHLBI ARDS Network. Subphenotypes in acute respiratory distress syndrome: latent class analysis of data from two randomised controlled trials. Lancet Respir. Med..

[5] Ben-Noun L (2000). Drug-induced respiratory disorders: incidence, prevention and management. Drug Saf..

[6] Lee-Chiong T, Matthay RA (2004). Drug-induced pulmonary edema and acute respiratory distress syndrome. Clin. Chest. Med..

[7] Dhokarh R (2012). Drug-associated acute lung injury: a population-based cohort study. Chest.

[8] Camus P, Fanton A, Bonniaud P, Camus C, Foucher P (2004). Interstitial lung disease induced by drugs and radiation. Respiration.

[9] Anan K (2017). Clinical characteristics and prognosis of drug-associated acute respiratory distress syndrome compared with non-drug-associated acute respiratory distress syndrome: a single-centre retrospective study in Japan. BMJ Open.

[10] Gibelin A (2016). Acute respiratory distress syndrome mimickers lacking common risk factors of the Berlin definition. Intensive Care Med..

[11] Gando S (2013). Japanese Association for Acute Medicine Disseminated Intravascular Coagulation (JAAM DIC) Study Group for the JAAM DIC Antithrombin Trial (JAAMDICAT). A randomized, controlled, multicenter trial of the effects of antithrombin on disseminated intravascular coagulation in patients with sepsis. Crit. Care.

[12] Ichikado K (2012). Fibroproliferative changes on high-resolution CT in the acute respiratory distress syndrome predict mortality and ventilator dependency: a prospective observational cohort study. BMJ Open.

[13] Kubo K (2013). Japanese Respiratory Society Committee for formulation of Consensus statement for the diagnosis and treatment of drug-induced lung injuries. Consensus statement for the diagnosis and treatment of drug-induced lung injuries. Respir. Investig..

[14] Thille AW (2013). Comparison of the Berlin definition for acute respiratory distress syndrome with autopsy. Am. J. Respir. Crit. Care Med..

[15] Kawamura K (2016). Efficacy of azithromycin in sepsis-associated acute respiratory distress syndrome: a retrospective study and propensity score analysis. SpringerPlus.

[16] Takaki M, Ichikado K, Kawamura K, Gushima Y, Suga M (2017). The negative effect of initial high-dose methylprednisolone and tapering regimen for acute respiratory distress syndrome: a retrospective propensity matched cohort study. Crit. Care.

[17] Kawamura K (2018). Adjunctive therapy with azithromycin for moderate and severe acute respiratory distress syndrome: a retrospective, propensity score-matching analysis of prospectively collected data at a single center. Int. J. Antimicrob. Agents.

[18] Anan K, Kawamura K, Suga M, Ichikado K (2018). Clinical differences between pulmonary and extrapulmonary acute respiratory distress syndrome: a retrospective cohort study of prospectively collected data in Japan. J. Thorac. Dis..

[19] Bernard GR (1994). The American-European Consensus Conference on ARDS. Definitions, mechanisms, relevant outcomes, and clinical trial coordination. Am. J. Respir. Crit. Care Med..

[20] ARDS Definition Task Force, Ranieri, V.M. (2012). Acute respiratory distress syndrome: the Berlin definition. JAMA..

[21] Moons KG (2015). Transparent Reporting of a multivariable prediction model for Individual Prognosis Or Diagnosis (TRIPOD): explanation and elaboration. Ann. Intern. Med..

[22] Ichikado K (2006). Prediction of prognosis for acute respiratory distress syndrome with thin-section CT: validation in 44 cases. Radiology.

[23] Gando S (2006). Japanese Association for Acute Medicine Disseminated Intravascular Coagulation (JAAM DIC) Study Group. A multicenter, prospective validation of disseminated intravascular coagulation diagnostic criteria for critically ill patients: comparing current criteria. Crit. Care Med..

[24] McCabe WR, Jackson GG (1962). Gram negative bacteremia: I. Etiology and ecology. Arch. Intern. Med..

[25] Harrell F (1996). Tutorial in Biostatistics multivariable prognostic models: Issues in developing models, evaluating assumptions and adequacy, and measuring and reducing errors. Stat. Med..

[26] Pencina MJ (2008). Evaluating the added predictive ability of a new marker: from area under the ROC curve to reclassification and beyond. Stat. Med..

